# Smart glasses integrated with a wireless ultrasound system for radial artery catheterization in elderly patients: a randomized controlled trial

**DOI:** 10.3389/fmed.2026.1884099

**Published:** 2026-07-23

**Authors:** Wudong Zhuang, Ruizhao Lyu, Xiaoyuan Li, Yang Bai, Rui Liu, Jianhua Wang

**Affiliations:** Department of Anesthesiology and Perioperative Medicine, Hebei Cangzhou Hospital of Integrated Traditional Chinese Medicine and Western Medicine, Hebei Province Key Laboratory of Integrated Traditional and Western Medicine in Neurological Rehabilitation, Hebei Key Laboratory of Integrated Traditional and Western Medicine in Osteoarthrosis Research (Preparing), Cangzhou, Hebei, China

**Keywords:** first puncture success rate, hand–eye coordination, portable wireless ultrasound, radial artery catheterization, smart glasses, ultrasound-guided

## Abstract

**Background:**

Radial artery catheterization can be challenging in elderly patients. The clinical value of integrated system combining a wireless ultrasound probe and smart glasses remains unclear. This study evaluated whether this approach improves first puncture success compared with conventional ultrasound guidance.

**Materials and methods:**

This prospective randomized controlled trial enrolled 194 patients aged ≥65 years with American Society of Anesthesiologists physical status I–III who were scheduled for elective surgery requiring radial artery catheterization. Patients were randomly assigned to the smart glasses group or conventional ultrasound group in a 1:1 ratio. The primary outcome was the first puncture success rate. Secondary outcomes included procedure-related behavioral indicators of hand–eye coordination, procedure time, and operator satisfaction.

**Results:**

A total of 194 patients were analyzed. The first puncture success rate was significantly higher in the smart glasses group than in the ultrasound group [88/97, 90.72% vs. 66/97, 68.04%; *p* < 0.001; relative risk (RR) = 1.333; 95% confidence interval (CI), 1.147–1.550]. Compared with the ultrasound group, the smart glasses group showed fewer head rotations [0 (0, 0) vs. 2 (1, 3); *p* < 0.001], ultrasound probe repositionings [0 (0, 1) vs. 1 (0, 2); *p* < 0.001], and needle redirections [2 (0, 2) vs. 2 (1, 3); *p* < 0.001]. Operator satisfaction, measured using a visual analog scale (VAS), was higher in the smart glasses group than in the ultrasound group [86 (79–90) vs. 70 (60–79); median difference, 13.00; 95% CI, 10.00–17.00; *p* < 0.001].

**Conclusion:**

Compared with conventional ultrasound guidance, integrated wireless ultrasound and smart glasses system improved the first puncture success rate of radial artery catheterization in elderly patients and enhanced procedure-related behavioral indicators associated with hand–eye coordination, including head rotations, ultrasound probe repositioning, and needle angle adjustments. This approach also shortened procedure time and improved operator satisfaction.

## Introduction

Radial artery catheterization is a routine and essential procedure in anesthesia and critical care for continuous blood pressure monitoring and arterial blood sampling (1–3). However, in elderly patients, physiological changes such as arteriosclerosis, vascular tortuosity, and luminal narrowing substantially increase procedural difficulty, resulting in lower catheterization success rates and higher risk of complications (3, 4).

Ultrasound guidance has been shown to significantly improve the accuracy and safety of arterial puncture and catheterization and has become the standard technique for enhancing procedural precision and safety (4–6). Multiple systematic reviews and meta-analyses consistently indicate that, compared with the conventional palpation method, ultrasound-guided radial artery catheterization significantly increases the first-pass success rate and reduces the number of puncture attempts as well as the incidence of complications such as hematoma (3, 4, 7–10).

Despite the clear advantages of ultrasound guidance, conventional console ultrasound systems are large, have complex cabling, and are limited in portability and maneuverability (11). Moreover, during the procedure, operators must frequently shift their attention among the patient, the ultrasound machine, and the puncture site, substantially increasing cognitive and physical workload, which particularly limits procedural efficiency in bedside or mobile settings (12).

Smart glasses technology can address the limitations of conventional ultrasound guidance by integrating imaging with the operator’s visual field, thereby reducing efficiency loss caused by frequent shifts in gaze. This approach is particularly suitable for bedside procedures, emergency settings, and anatomically challenging punctures. In recent years, the integration of smart glasses with ultrasound technology has attracted widespread attention in the medical field and holds great potential for future applications (12–20).

Multiple studies have shown that smart glasses can improve puncture success rates and operator satisfaction by enhancing hand–eye coordination through reducing the operator’s head movements (12, 14, 18, 21). In studies conducted on adult patients, smart glasses have been shown to significantly increase the first-pass success rate of ultrasound-guided radial artery catheterization, reduce frequent head movements between the surgical field and the ultrasound screen, thereby streamlining the procedure, shortening operation time, and enhancing physician satisfaction (12). These positive findings have also been validated in pediatric patients (14, 18). These study results indicate that smart glasses offer significant advantages in improving the success rate and procedural efficiency of ultrasound-guided radial artery puncture.

However, whether smart glasses combined with ultrasound can truly improve puncture success rates remains inconclusive and requires further investigation. A study by Gözen et al. showed that smart glasses did not significantly increase the first-pass success rate of radial artery puncture. Previous research has also indicated that the use of smart glasses with ultrasound guidance for peripheral venous puncture did not significantly improve first-pass success rates or reduce the number of puncture attempts (16, 17). A prospective randomized controlled trial investigated the use of integrated wireless ultrasound and smart glasses system for radial artery puncture; however, it still relied on a tablet for image transmission, indicating that the system could be further simplified (13). To date, no studies have investigated the effect of an integrated wireless ultrasound and smart glasses system on the first-pass success rate of radial artery catheterization in elderly patients.

The primary aim of this study was to evaluate the effect of an integrated wireless ultrasound and smart glasses system on the first-pass success rate of radial artery catheterization in elderly patients. The secondary aims were to assess hand–eye coordination using procedure-related behavioral indicators, including head rotations, ultrasound probe repositionings, and needle redirections, as well as total number of puncture attempts, overall success rate, procedural difficulty.

## Methods

### Study design and participants

This study was a single-center, prospective, randomized controlled trial designed to evaluate whether the use of integrated wireless ultrasound and smart glasses system could improve the first puncture success rate, reduce procedure-related behavioral indicators associated with hand–eye coordination, and increase operator satisfaction during radial artery catheterization in elderly patients. This randomized controlled trial was conducted in the Department of Anesthesiology and Perioperative Medicine at Cangzhou Hospital of Integrated Traditional Chinese and Western Medicine, Hebei Province (a tertiary A-level hospital). This study was approved by the Ethics Committee (Approval No.: CZX2025-KY030-01; Date of Approval: May 6, 2025) and registered in the Chinese Clinical Trial Registry prior to patient enrollment.^1^ The trial was conducted at Cangzhou Hospital of Integrated Traditional Chinese and Western Medicine, Hebei Province, from June 10, 2025, to April 28, 2026, in strict accordance with the Consolidated Standards of Reporting Trials (CONSORT). No protocol modifications were made after the trial commenced. All participants provided written informed consent.

This study included patients of any sex who were scheduled for elective surgery and planned to undergo radial artery catheterization. Inclusion criteria were: patient or family consent via signed informed consent, age ≥65 years, and American Society of Anesthesiologists (ASA) physical status I–III. Exclusion criteria were: recent radial artery puncture, presence of a wound or infected hematoma at the catheterization site, peripheral vascular disease, coagulation disorders, inadequate collateral circulation (Allen’s test negative), unstable vital signs, severe arrhythmias, or shock. Eligible patients had their radial artery depth and diameter measured in the preoperative preparation room prior to catheterization.

### Randomization and blinding

An independent researcher, who was not involved in patient enrollment or clinical procedures, prepared 194 sequentially numbered opaque, sealed envelopes using a random number generator. Each envelope contained a card with a random number and group assignment, and the envelopes were securely stored in numerical order. Patients were randomly assigned in a 1:1 ratio to either the smart glasses group or the conventional ultrasound group. After confirming eligibility and obtaining informed consent, a trained research nurse opened the envelope corresponding to each patient immediately prior to radial artery puncture and informed the operator of the group assignment. The research nurse responsible for envelope management did not participate in data collection or outcome assessment.

Due to the nature of the intervention, participants were blinded to group assignment, whereas the operators could not be blinded. In both groups, radial artery catheterization was performed preoperatively by one of three attending anesthesiologists who were proficient in ultrasound-guided radial artery catheterization (confirmed via written questionnaire). All anesthesiologists were right-handed, board-certified, and experienced in ultrasound-guided radial artery catheterization using the short-axis out-of-plane (SA-OOP) technique, each having performed more than 100 such procedures. The operators had no prior experience using the integrated wireless ultrasound and smart glasses system for ultrasound-guided radial artery catheterization. All three anesthesiologists received standardized knowledge and procedural training before the study commenced. Before study initiation, each anesthesiologist completed at least 50 ultrasound-guided radial artery catheterization procedures using the integrated wireless ultrasound and smart glasses system during standardized training to achieve procedural proficiency. During the trial, after patient-level randomization to the smart glasses or conventional ultrasound group, procedures were assigned to the three anesthesiologists using a block-balanced operator allocation scheme. Within each block of three consecutive procedures, the three operators were randomly ordered so that each operator performed one procedure per block whenever clinical scheduling allowed. This approach was intended to preserve randomness while keeping the number of procedures performed by each operator as balanced as possible across study groups, thereby reducing potential operator-related selection bias. After each procedure, the operator completed a post-procedure questionnaire. The operators did not participate in data analysis or manuscript preparation.

Behavioral indicators, including head rotations, ultrasound probe repositionings, and needle redirections, were prospectively recorded in real time by a designated investigator using predefined operational definitions and standardized case report forms. Synchronized video recordings of both the operator’s head movements and the procedural field were obtained throughout each procedure. Because the intervention was visually distinguishable, blinding of the behavioral assessor to group allocation was not feasible. To improve measurement reliability, all behavioral outcomes were independently reviewed and verified by a second investigator using the synchronized video recordings as the reference source according to the predefined operational definitions. Any discrepancies between the real-time records and video review were resolved by consensus before final data entry. The synchronized video recordings served as the reference source for confirmation of behavioral events.

However, the researchers responsible for measuring radial artery depth and diameter, as well as assessing complications and catheter function, were blinded to group allocation. To minimize measurement bias, radial artery depth and diameter were independently measured by two investigators who were blinded to group allocation, and the average value was used for statistical analysis.

### Study procedures

In the preoperative preparation room, patients underwent continuous monitoring of electrocardiography, noninvasive blood pressure, and oxygen saturation, and an intravenous line was established. Prior to the procedure, dexmedetomidine was administered via infusion pump at a loading dose of 3 μg·kg^−1^·h^−1^, followed by a maintenance dose of 0.3 μg·kg^−1^·h^−1^ after 10 min. Sufentanil citrate 5–10 μg was administered intravenously, and oxygen was delivered via face mask at a flow rate of 4 L/min. After randomization, the puncture side (left or right) was selected according to the type of surgery and surgical position. Patients were placed in the supine position with the forearm abducted at 90°, a thin pillow under the wrist, palm facing upward, and the dorsum of the hand extended and secured. The ultrasound probe was coated with coupling gel and covered with a sterile sheath. Radial artery depth and diameter were independently measured by two blinded researchers, and the average value was recorded. The operator adjusted the ultrasound probe’s depth and gain for each patient to achieve optimal imaging, and positioned the probe to center the radial artery on the screen. Color Doppler ultrasound was used to assess vascular patency and detect any abnormalities. Once unobstructed blood flow and absence of vascular anomalies were confirmed, standard disinfection and draping were performed. Local infiltration anesthesia was administered using 2% lidocaine. A 20G BRAUN B indwelling needle was used for puncture in both groups.

### Ultrasound group (control group)

**Figure 1:**
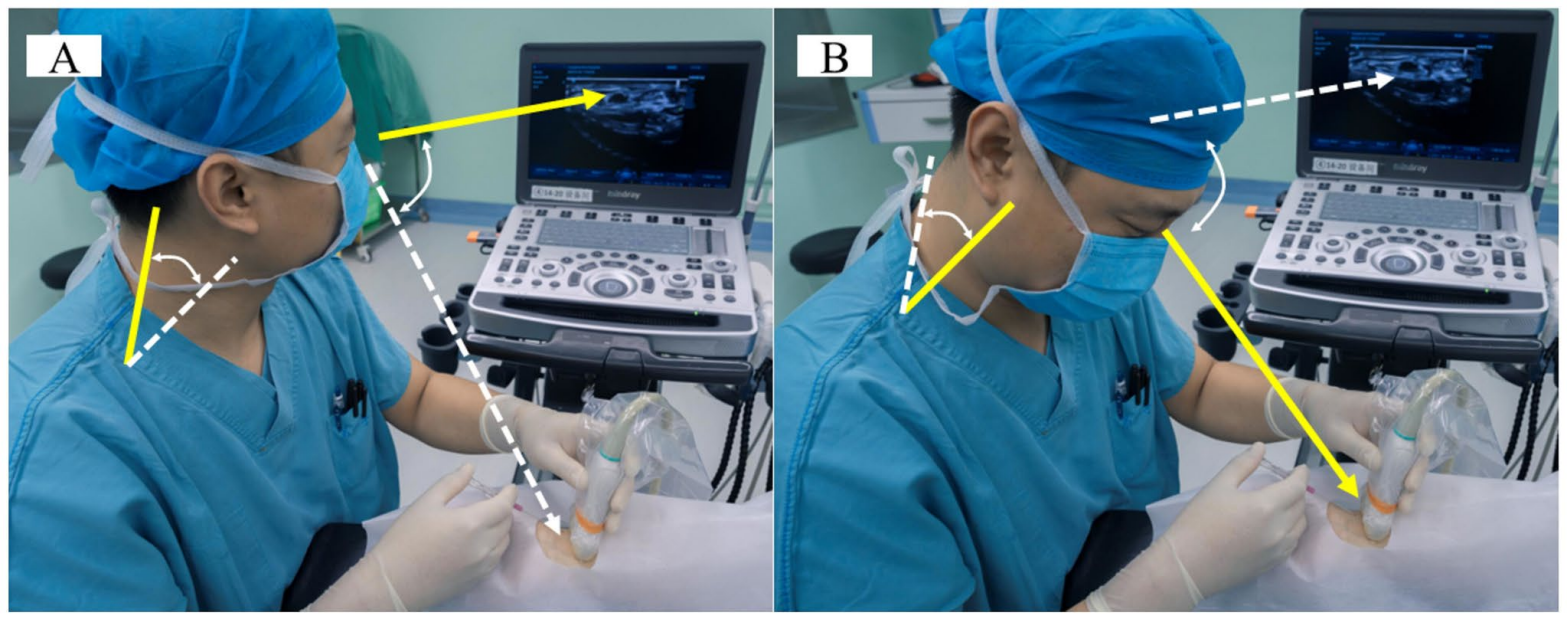
Operator gaze and posture during conventional ultrasound-guided radial artery catheterization. **(A)** The operator turns the head to view the ultrasound monitor. **(B)** The operator returns gaze to the puncture site during needle advancement.

In the ultrasound group, operators performed radial artery catheterization under ultrasound guidance (Figure 1). The ultrasound machine was consistently placed on the upper arm side near the radial artery puncture site, and operators were seated while using the short-axis out-of-plane (SA-OOP) dynamic needle-tip technique (3, 22). A high-frequency linear ultrasound probe (L12-4s) of a Mindray medical ultrasound system (UMT-500, Shenzhen Mindray Bio-Medical Electronics Co., Ltd.) was used and covered with a disposable sterile sheath. The probe depth and gain were individually adjusted, and the probe position was optimized to center the radial artery on the screen. Prior to needle insertion, the probe orientation was verified to ensure that the displayed image corresponded accurately with the correct anatomical position (23). The operator, seated, held the arterial puncture needle in the right hand, with the needle shaft angled at 30–45° to the skin, and inserted it at the intersection of the ultrasound probe and the skin. Under ultrasound guidance, the hyperechoic bright spot at the needle tip was advanced into the radial artery lumen. After depressing the needle hub, the needle was further advanced 1–2 mm. Once smooth blood return was observed, the catheter was advanced and the needle stylet withdrawn. The catheter was connected to an arterial pressure monitoring system, and successful catheterization was confirmed by the appearance of an arterial pressure waveform, followed by dressing and securing the catheter. If catheterization failed after three attempts or if complications required changing the puncture site, the procedure was considered unsuccessful, and puncture was performed on the contralateral radial artery or the dorsalis pedis artery. Postoperatively, ultrasound was used to assess radial artery depth, diameter, and procedural complications (e.g., vasospasm, hematoma). For all patients, ultrasound localization time, radial artery puncture and catheterization duration, ergonomic parameters, anesthesia- and puncture-related complications, and arterial catheter function were recorded and evaluated.

### Smart glasses group (experimental group)

**Figure 2:**
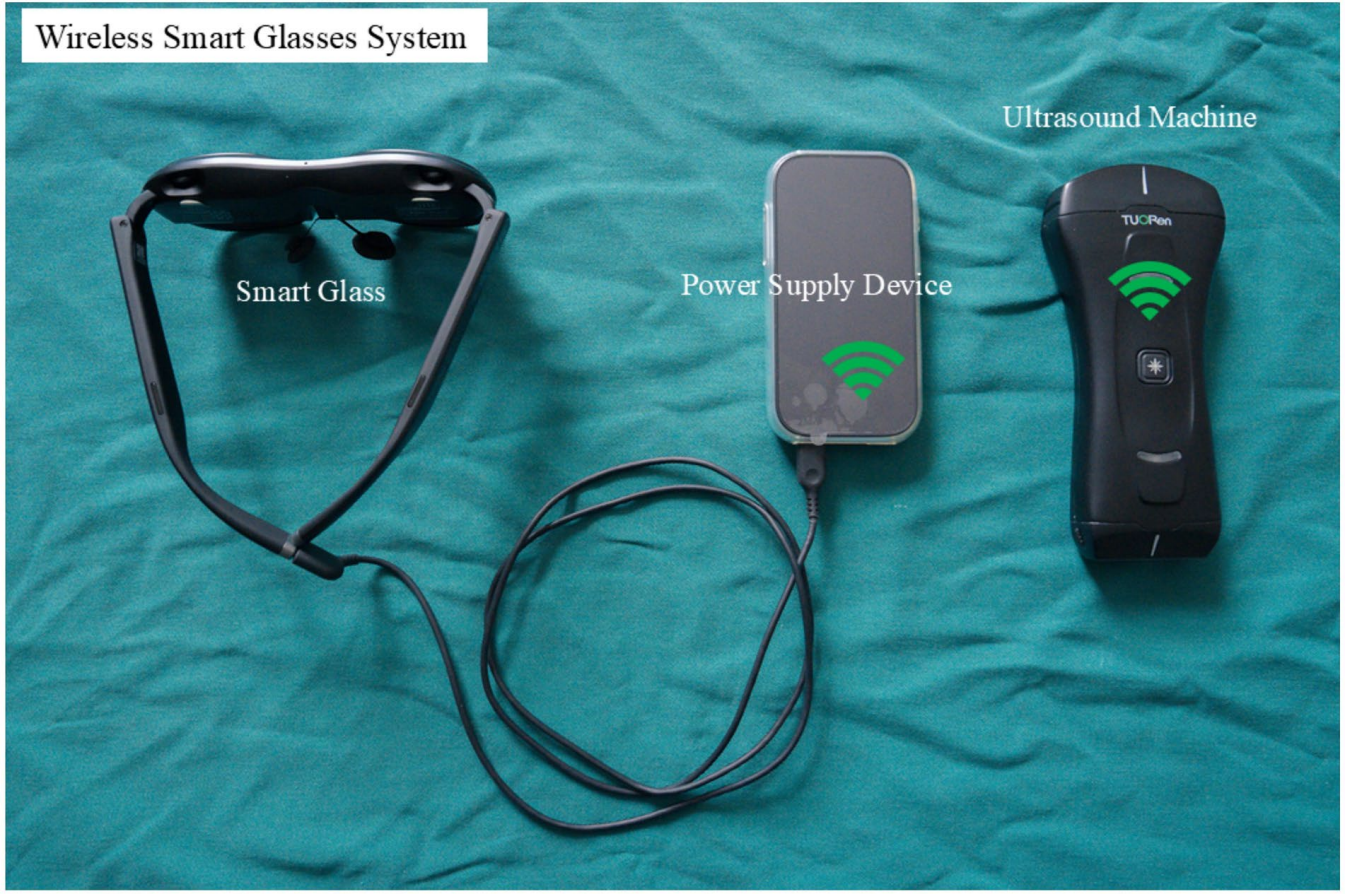
Integrated wireless ultrasound and smart glasses system setup.

A TUORen TR-WP1 wireless handheld color ultrasound device (Zhengzhou TUORen Medical Equipment Co., Ltd.) with a high-frequency linear probe (7.5–10 MHz) covered with a disposable sterile sheath was used. The ultrasound probe was wirelessly connected to the ROKID Station2 portable AR console, which in turn was connected to the smart glasses (Rokid Max 2, Hangzhou Lingban Technology Co., Ltd. d, Hangzhou, China) via a full-feature Type-C data cable, allowing real-time projection of the ultrasound image into the operator’s visual field (Figure 2). The smart glasses were equipped with a diopter adjustment knob on the top of the frame, with a range of 0–600 diopters, allowing operators who wore corrective lenses to use them. During use, the ROKID Station2 continuously supplied power to the smart glasses and transmitted ultrasound images. In the smart glasses group, operators required approximately 5 s before the procedure to don and adjust the smart glasses.

**Figure 3:**
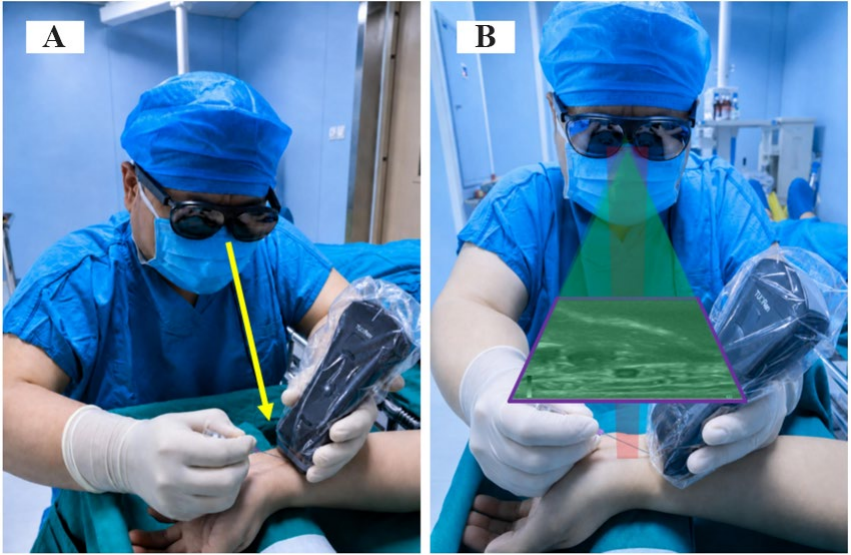
System-based ultrasound-guided radial artery catheterization. **(A)** The operator views the puncture site while holding the wireless ultrasound probe. **(B)** The smart glasses project the real-time ultrasound image into the operator’s visual field.

During puncture, the operator directly viewed the ultrasound images projected by the smart glasses, eliminating the need to frequently shift the head and gaze between the patient and the ultrasound screen. The puncture could be observed through the gap beneath the display device (Figure 3). After the procedure, the smart glasses and wireless ultrasound device were returned to their designated positions and managed and disinfected by trained personnel. All other procedural steps—including anesthesia, puncture technique, catheterization confirmation, and complication assessment—were identical to those in the control group.

### Outcome measurements

#### Primary outcome

The first puncture success rate of the radial artery catheter, defined as successful insertion of the arterial catheter with a single skin puncture and confirmation by the presence of an arterial pressure waveform displayed on the electrocardiographic monitor.

#### Secondary outcomes included

(1) Hand–eye coordination: number of head rotations (defined as head movement exceeding 45°, including flexion or rotation), number of ultrasound probe repositionings (defined as moving the probe to recapture the needle or target vessel), and number of needle redirections (defined as withdrawing the needle, adjusting its direction, or reinserting at a different angle); (2) Overall success rate: defined as successful catheterization of the selected radial artery within no more than three skin punctures; (3) Total number of attempts: defined as the total number of skin punctures performed to achieve successful radial artery catheterization. An attempt was counted each time the needle breached the skin. Withdrawal of the needle followed by reinsertion was considered a new attempt, whereas minor needle manipulations without full withdrawal were not counted as separate attempts. Failed attempts were included in the total count; (4) Ultrasound localization time: defined as the duration from probe placement to the first skin puncture; (5) Catheterization time: defined as the interval from the first skin puncture to the appearance of an arterial pressure waveform, independent of the number of catheterization attempts; (6) Radial artery diameter (defined as the inner diameter) and radial artery depth (defined as the perpendicular distance from the anterior vessel wall to the skin surface); (7) Overall complication rate: including hematoma (defined as a hypoechoic area around the vessel detected by ultrasound) and vasospasm (defined as a ≥ 25% reduction in vessel diameter after catheterization in the absence of an intravascular hematoma) (24), arterial thrombosis (defined as abnormal intravascular echoes detected by ultrasound or impaired distal hand circulation) and catheter malfunction (defined as loss of catheter function, absence of blood return, disappearance of the arterial waveform, or catheter displacement, with inability to monitor or obtain blood even after flushing the catheter or changing the dressing) (14, 25); (8) To evaluate musculoskeletal fatigue and operator-reported outcomes during the procedure, the Operators’ Ergonomic Fatigue Score was used to assess fatigue and pain in the neck, shoulders, waist, eyes, and upper limbs, which were graded into three levels: no pain, mild pain, and moderate-to-severe pain. Operator satisfaction was defined as the operator’s overall subjective evaluation of the procedure, incorporating perceived procedural ease, visual–motor coordination efficiency, and overall workflow during radial artery catheterization. Operator satisfaction and procedural difficulty were assessed using a 100 mm Visual Analog Scale (VAS), where 100 indicated the highest level of satisfaction and 0 indicated complete dissatisfaction. The satisfaction scale was measured using an unmarked 10 cm ruler (12, 14, 19). Procedural difficulty was defined as the operator’s subjective assessment of the technical complexity of the procedure, taking into account factors such as vessel visualization, needle control, the need for repeated adjustments, and overall procedural fluency. Procedural difficulty was categorized as easy, moderate, or difficult.

### Sample size calculation

Based on relevant literature, the first puncture success rates in the smart glasses group and the ultrasound-guided group were 88.3 and 72.1%, respectively (12). Using a two-sided test with a significance level of *α* = 0.05, a power (1–*β*) of 0.8, and a 1:1 allocation ratio, the calculated sample size was 92 patients per group. After adjusting for a 5% potential dropout rate, the final sample size was set at 97 patients per group.

### Statistical analysis

Continuous variables were assessed for normality using the Kolmogorov–Smirnov test and visual inspection of histograms. Normally distributed continuous variables are presented as mean ± standard deviation and were compared using the independent-samples t test. Non-normally distributed continuous variables are presented as median [interquartile range, IQR] and were compared using the Mann–Whitney U test. Median differences with 95% confidence intervals were estimated using the Hodges–Lehmann method where appropriate for non-normally distributed variables.

Total number of attempts was approximately normally distributed and therefore treated as a continuous variable. It was analyzed using an independent-samples *t* test and reported as mean difference with 95% confidence interval. For count-based outcomes with heavily tied distributions, where non-parametric Hodges–Lehmann estimates may become unstable and yield uninformative confidence intervals, parametric mean-based estimates were preferentially reported when distributional assumptions were satisfied.

Categorical variables are presented as *n* (%) and were compared using the chi-square test or Fisher’s exact test, as appropriate. The primary outcome, first puncture success rate, was compared between groups using the chi-square test, and relative risks with 95% confidence intervals were reported. Ordinal questionnaire outcomes, including operator satisfaction, procedural difficulty, and ergonomic fatigue scores, were compared using the Mann–Whitney U test. Category-specific relative risks with 95% confidence intervals for questionnaire response categories were presented as descriptive effect estimates. All statistical tests were two-sided, and a *p* value < 0.05 was considered statistically significant. Statistical analyses were performed using IBM SPSS Statistics version 27.0 (IBM Corp., Armonk, NY, United States).

## Results

**Figure 4:**
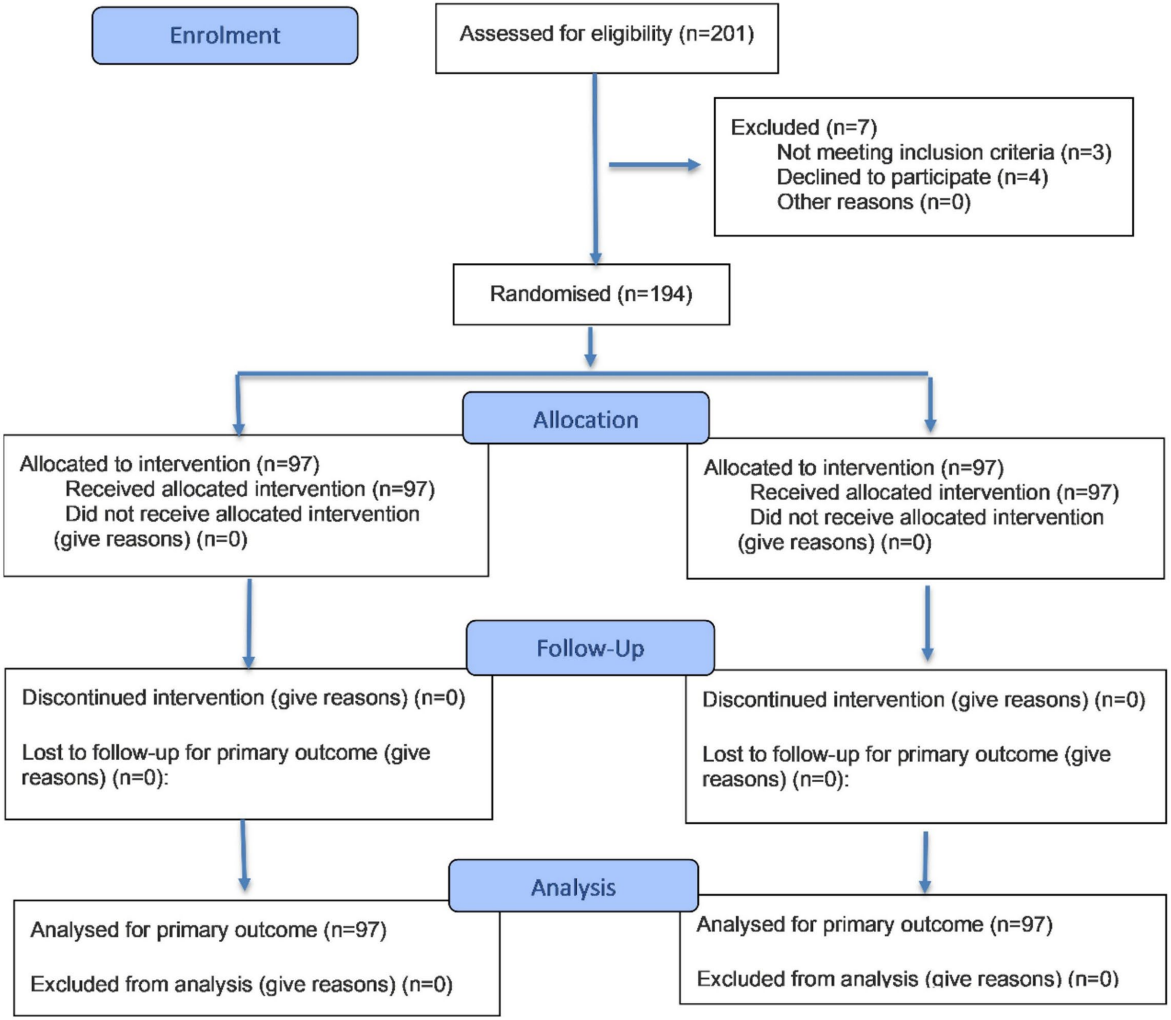
CONSORT 2025 flow diagram: Flow diagram of the progress through the phases of a randomized trial of two groups (that is, enrolment, intervention allocation, follow-up, and data analysis).

A total of 201 patients were initially assessed for eligibility, of whom 7 were excluded before randomization, including 3 patients who did not meet the inclusion criteria and 4 who declined to participate. Ultimately, 194 patients were enrolled and randomly assigned in a 1:1 ratio to the smart glasses group (*n* = 97) or the ultrasound group (*n* = 97), and all patients completed the study procedures. The study was conducted in accordance with the CONSORT guidelines (Figure 4) in the Department of Anesthesiology and Perioperative Medicine at Cangzhou Hospital of Integrated Traditional Chinese and Western Medicine, Hebei Province, from June 10, 2025, to April 28, 2026. No protocol violations were reported during the study period, and complete data for both primary and secondary outcomes were obtained without missing values.

### Baseline characteristics

There were no significant differences between the two groups in age, sex, height, weight, body mass index (BMI), underlying diseases, ASA physical status, pre-catheterization radial artery depth, or radial artery diameter (Table 1).

**Table 1:** Baseline characteristics of the patients (*n* = 194).

Characteristic	Smart glasses group (*n* = 97)	Control group (*n* = 97)	*p* value
Age (yr), median [IQR]	72.00 [69.00, 79.00]	71.00 [68.00, 79.00]	0.471
Sex, *n* (%)			0.565
Male	50 (51.55%)	54 (55.67%)	
Female	47 (48.45%)	43 (44.33%)	
Height (cm), mean ± SD	155.94 ± 7.07	156.66 ± 7.03	0.477
Weight (kg), mean ± SD	67.32 ± 8.25	67.00 ± 8.46	0.790
BMI (kg/m^2^), mean ± SD	27.82 ± 4.01	27.40 ± 3.80	0.451
Underlying diseases, *n* (%)			
Hypertension	51 (52.58%)	52 (53.61%)	0.886
Coronary artery disease	19 (19.59%)	22 (22.68%)	0.598
Diabetes mellitus	24 (24.74%)	27 (27.84%)	0.625
Pulmonary disease	10 (10.31%)	11 (11.34%)	0.817
Arrhythmia	10 (10.31%)	13 (13.40%)	0.505
ASA physical status, *n* (%)			0.772
II	54 (55.67%)	56 (57.73%)	
III	43 (44.33%)	41 (42.27%)	
Pre-catheterization radial artery depth (mm), median [IQR]	2.30 [2.00, 2.65]	2.30 [1.90, 2.60]	0.998
Pre-catheterization radial artery diameter (mm), median [IQR]	1.90 [1.70, 2.10]	1.90 [1.60, 2.10]	0.231
Side of radial artery, *n* (%)			1.000
Right	52 (53.61%)	52 (53.61%)	
Left	45 (46.39%)	45 (46.39%)	

BMI, Body Mass Index; ASA, American Society of Anesthesiologists; IQR, Interquartile Range; SD, Standard deviation. Continuous variables are expressed as mean ± standard deviation or median [IQR], and categorical variables are expressed as *n* (%).

### Primary outcome

The first puncture success rate of radial artery catheterization was significantly higher in the smart glasses group (88/97, 90.72%) than in the ultrasound group (66/97, 68.04%) [*p* < 0.001; relative risk (RR) = 1.333; 95% confidence interval (CI), 1.147–1.550] (Table 2).

**Table 2:** Comparison of radial artery catheterization outcomes between the control group and the smart glasses group.

Characteristic	Smart glasses group (*n* = 97)	Control group (*n* = 97)	Effect size (95% CI)	*p* value
Primary outcome				
First puncture success rate	88 (90.72%)	66 (68.04%)	RR = 1.333 (1.147, 1.550)	< 0.001^χ^
Secondary outcomes				
Hand–eye coordination				
Ultrasound probe repositionings	0.00 [0.00, 1.00]	1.00 [0.00, 2.00]	Median difference = 0.000 (−1.000, 0.000)	< 0.001^U^
Needle redirections	2.00 [0.00, 2.00]	2.00 [1.00, 3.00]	Median difference = −1.000 (−1.000, 0.000)	< 0.001^U^
0 redirections, *n* (%)	31 (31.96%)	11 (11.34%)		
1 redirections, *n* (%)	14 (14.43%)	16 (16.49%)		
2 redirections, *n* (%)	30 (30.93%)	23 (23.71%)		
≥ 3 redirections, *n* (%)	22 (22.68%)	47 (48.45%)		
Head rotations	0.00 [0.00, 0.00]	2.00 [1.00, 3.00]	Median difference = −2.000 (−2.000, −2.000)	< 0.001^U^
Overall success rate	92 (94.85%)	82 (84.54%)	RR = 1.122 (1.018, 1.236)	0.018^χ^
Total number of attempts	1.21 ± 0.735	1.67 ± 1.179	Mean difference = −0.464 (−0.742, −0.186)	0.001^T^
1 attempt, *n* (%)	88 (90.72%)	66 (68.04%)		
2 attempt, *n* (%)	4 (4.12%)	15 (15.46%)		
3 attempt, *n* (%)	0	2 (2.06%)		
4 attempt, *n* (%)	4 (4.12%)	10 (10.31%)		
5 attempt, *n* (%)	1 (1.03%)	4 (4.12%)		
Ultrasound localization time (s)	12.00 [10.00, 15.00]	16.00 [13.00, 19.00]	Median difference = −3.000 (−4.000, −2.000)	< 0.001^U^
Catheterization time (s)	23.00 [21.00, 26.00]	30.00 [27.00, 36.00]	Median difference = −7.000 (−8.000, −5.000)	< 0.001^U^
Total procedure time (s)	36.00 [32.00, 40.00]	46.00 [41.00, 55.00]	Median difference = −10.000 (−12.000, −8.000)	< 0.001^U^
Overall adverse events	8 (8.25%)	21 (21.65%)	RR = 0.381 (0.177, 0.818)	0.009^χ^
Hematoma	8 (8.25%)	14 (14.43%)	RR = 0.571 (0.251, 1.300)	0.174^F^
Vasospasm	1 (1.03%)	1 (1.03%)	N/A	N/A
Arterial thrombosis	0	0	N/A	N/A
Catheter malfunction	1 (1.03%)	7 (7.22%)	RR = 0.143 (0.018, 1.139)	0.065^F^
Post-catheterization radial artery depth (mm)	2.20 [2.00, 2.60]	2.30 [2.00, 2.55]	Median difference = 0.000 (−1.000, 0.200)	0.631^U^
Post-catheterization radial artery diameter (mm)	1.90 [1.70, 2.10]	1.90 [1.60, 2.00]	Median difference = 0.1000 (0.000, 0.100)	0.191^U^

Overall adverse events were counted at the patient level. Individual adverse events were recorded separately; therefore, one patient could contribute to more than one adverse event category. In the smart glasses group, two patients experienced multiple concurrent adverse events, and in the control group, one patient experienced multiple concurrent adverse events. Continuous variables are expressed as mean ± standard deviation (SD) or median [interquartile range (IQR)], as appropriate. Categorical variables are presented as number (percentage). CI, Confidence interval; IQR, Interquartile range; SD, Standard deviation; N/A, Not applicable. χ, chi-square test; U, Mann–Whitney U test; F, Fisher’s exact test; T, independent-samples *t*-test.

### Secondary outcomes

Secondary outcomes are reported as exploratory analyses because no formal adjustment for multiple comparisons was applied.

Indicators of hand–eye coordination were significantly improved in the smart glasses group, as reflected by fewer head rotations [0 (0, 0) vs. 2 (1, 3), *p* < 0.001; median difference −2; 95% CI − 2 to −2], number of ultrasound probe repositionings [0 (0, 1) vs. 1 (0, 2), *p* < 0.001; median difference 0; 95% CI − 1 to 0], and number of needle redirections [2 (0, 2) vs. 2 (1, 3), *p* < 0.001; median difference −1; 95% CI − 1 to 0] (Table 2).

The overall success rate was significantly higher in the smart glasses group than in the ultrasound group (92/97, 94.85% vs. 82/97, 84.54%; *p* = 0.018; RR = 1.122, 95% CI 1.018–1.236). The total number of attempts was significantly lower in the smart glasses group compared with the control group (1.21 ± 0.74 vs. 1.67 ± 1.18, *p* = 0.001; mean difference = −0.464, 95% CI − 0.742 to −0.186; Table 2).

The ultrasound localization time was significantly shorter in the smart glasses group than in the ultrasound group [12 (10, 15) s vs. 16 (13, 19) s, *p* < 0.001; median difference −3; 95% CI − 4 to −2]. Catheterization time was also significantly shorter in the smart glasses group [23 (21, 26) s vs. 30 (27, 36) s, *p* < 0.001; median difference −7; 95% CI − 8 to −5]. In addition, the total procedure time was significantly shorter in the smart glasses group than in the ultrasound group [36 (32, 40) s vs. 46 (41, 55) s, *p* < 0.001; median difference −10; 95% CI − 12 to −8] (Table 2).

The overall incidence of adverse events was significantly lower in the smart glasses group than in the ultrasound group (8/97, 8.25% vs. 21/97, 21.65%; *p* = 0.009; RR = 0.381, 95% CI 0.177–0.818). There were no significant differences between the two groups in the incidence of hematoma, vasospasm, arterial thrombosis, or catheter malfunction. The overall adverse-event rate represented the number of patients who experienced at least one adverse event. Because some patients experienced more than one adverse event, the sum of individual adverse-event categories exceeded the overall number of affected patients. In addition, no significant differences were observed between the groups in post-catheterization radial artery depth or diameter (Table 2).

### Operator questionnaire assessment

Operator satisfaction scores were significantly higher in the smart glasses group than in the control group [86 (79, 90) vs. 70 (60, 79); median difference, 13.00; 95% CI, 10.00–17.00; *p* < 0.001]. The overall distributions of procedural difficulty and ergonomic fatigue scores also differed significantly between groups (both *p* < 0.001) (Table 3). The categorical distribution of satisfaction scores is provided in Supplementary Table S1.

**Table 3:** Comparison of operator questionnaire responses between the smart glasses group and the control group.

Characteristic	Smart glasses group (*n* = 97)	Control group (*n* = 97)	Effect size (95% CI)	*p* value
Satisfaction score	86 [79, 90]	70 [60, 79]	Median difference = 13.00 (10.00, 17.00)	< 0.001^U^
Procedural difficulty				< 0.001^U^
Easy	74 (76.29%)	28 (28.87%)	RR = 2.643 (1.897, 3.682)	
Moderate	14 (14.43%)	42 (43.30%)	RR = 0.333 (0.195, 0.569)	
Difficult	9 (9.28%)	27 (27.84%)	RR = 0.333 (0.166, 0.671)	
Ergonomic fatigue score				< 0.001^U^
No fatigue	67 (69.07%)	27 (27.84%)	RR = 2.481 (1.754, 3.511)	
Mild fatigue	20 (20.62%)	35 (36.08%)	RR = 0.571 (0.356, 0.916)	
Moderate-to-severe fatigue	10 (10.31%)	35 (36.08%)	RR = 0.286 (0.150, 0.544)	

Data are presented as median [IQR] or *n* (%). Satisfaction score was analyzed as a continuous VAS variable. Procedural difficulty and ergonomic fatigue score were analyzed as ordinal variables. Between-group differences were assessed using the Mann–Whitney U test. The effect estimate for satisfaction score is the Hodges–Lehmann median difference with 95% CI. Category-specific relative risks with 95% CIs are presented as descriptive effect estimates. VAS, visual analog scale; IQR, interquartile range; RR, relative risk; CI, confidence interval. ᵁMann–Whitney U test.

## Discussion

This study demonstrated that the use of an integrated wireless ultrasound and smart glasses system improved the success of radial artery catheterization in elderly patients, while also enhancing procedural efficiency, optimizing hand–eye coordination, reducing short-term procedure-related adverse events, and increasing operator satisfaction. These findings are consistent with previous studies (12, 14, 18).

Notably, this study is the first randomized controlled trial to investigate the use of integrated wireless ultrasound and smart glasses system for radial artery catheterization in elderly patients. The study population consisted of patients aged ≥65 years, a group with a high prevalence of arteriosclerosis, vascular tortuosity, and luminal narrowing. These vascular changes increase the technical difficulty of arterial puncture and catheterization.

During the procedure, radial artery catheterization was performed using the short-axis out-of-plane (SA-OOP) technique. In addition, a standardized ultrasound machine position was adopted based on the operators’ preferred ergonomic setup. These standardized procedural conditions were implemented to minimize bias related to individual operating habits (26). One of the major challenges of ultrasound-guided radial artery catheterization is that operators must not only possess detailed anatomical knowledge but also maintain coordinated interaction among hand movements, visual attention, the procedural field, and the ultrasound display (23). With conventional console ultrasound guidance, operators must repeatedly shift their gaze between the puncture site and the ultrasound screen. Frequent head and eye movements may lead to loss of visual orientation, delayed needle-tip localization, and impaired procedural coordination (3). The increased cognitive workload associated with repeated visual shifts may impair the operator’s ability to detect subtle imaging details, such as needle-tip echoes, in real time (3, 23). In contrast, the smart glasses projected the ultrasound image directly into the operator’s forward visual field, allowing closer alignment between ultrasound visualization and hand movements (15). This spatial integration may have reduced gaze shifting and improved procedural workflow, although cognitive workload was not directly measured in this study.

In this study, the first puncture success rate in the smart glasses group reached 90.72%, representing an increase of approximately 22.7 percentage points compared with 68.04% in the control group (RR = 1.333; 95% CI 1.147–1.550). The first puncture success rate in the control group (68.04%) was lower than that reported by Wang et al. for the adult control group (72.1%), whereas the rate in the smart glasses group (90.72%) exceeded their reported value (88.3%) (12). This study employed a wireless ultrasound probe design, eliminating the cable constraints associated with conventional ultrasound systems and substantially improving procedural flexibility. The first puncture success rate in the smart glasses group was therefore higher than that reported in the aforementioned studies. The present study enrolled elderly patients aged ≥65 years, whereas the study by Wang et al. included patients aged 18–70 years. Elderly patients have a higher prevalence of arteriosclerosis, vascular tortuosity, and luminal narrowing, all of which substantially increase puncture difficulty. This may explain why the first puncture success rate in the ultrasound group was lower than that reported by Wang et al. Not all studies, however, have demonstrated a significant improvement in the first puncture success rate with smart glasses. One randomized controlled trial showed that combining smart glasses with laser trajectory guidance significantly improved one-time puncture success, reduced the number of needle redirections, and shortened procedure time. The benefit was particularly evident among junior operators who were less familiar with the long-axis in-plane technique, suggesting that laser trajectory visualization may have a synergistic effect when combined with smart glasses, especially for less experienced users. These findings are consistent with the results of the present study and further support the concept that optimizing visual guidance and spatial alignment can improve the precision and efficiency of ultrasound-guided vascular puncture. In contrast, Gözen et al. reported that smart glasses did not significantly improve the first puncture success rate (13). Their study enrolled a general adult population, included a relatively small sample size, and reported a baseline first puncture success rate of 86.7% in the control group, leaving limited room for improvement. These findings suggest that the benefits of smart glasses may vary across different patient populations.

In this study, hand–eye coordination was assessed using three objective behavioral indicators: number of head rotations, number of ultrasound probe repositionings, and number of needle redirections. The smart glasses group showed significantly fewer head rotations than the control group (0 vs. 2, *p* < 0.001), which is consistent with findings from previous studies (12–14, 18). These measures were used as indirect indicators related to visuomotor workflow rather than as direct measurements of hand–eye coordination itself. Because projection of the ultrasound image into the operator’s forward visual field inherently reduces the need for head movement, head rotation should be interpreted as a workflow-related process indicator rather than an independent clinical outcome. Head rotation may transmit mechanical tension through the fascial chain to the shoulder girdle, thereby affecting the stability of probe handling (27). A reduction in head rotations not only decreases cervical fatigue but may also indirectly improve probe stability and needle insertion accuracy. The use of smart glasses reduced the number of ultrasound probe repositionings and needle redirections, allowing operators to observe the relationship between the needle tip and the vessel in real time and make fine adjustments at an early stage of deviation (16).

This study is the first to combine a wireless handheld color ultrasound probe with smart glasses, creating a truly portable, integrated puncture guidance system consisting of smart glasses, a portable AR console, and a wireless probe. This system offers three major advantages: First, it eliminates cable obstruction. In conventional portable ultrasound, probe cables restrict movement and workspace, obscure the visual field, and interfere with accurate needle-tip positioning. Second, it avoids tethering effects. Continuous tension from a wired probe increases the difficulty of maintaining stable hand control during the procedure. Third, it enhances procedural flexibility. The wireless probe is lightweight and free from console cabling constraints, making it especially suitable for bedside, mobile, and technically challenging puncture scenarios (17).

Procedural efficiency was significantly improved in the smart glasses group, with a median total procedure time reduced from 46 to 36 s. Both ultrasound localization time (12 vs. 16 s) and catheterization time (23 vs. 30 s) were also shorter compared to the control group. This improvement is likely attributable to several synergistic factors. First, continuous visual feedback enabled operators to maintain precise spatial awareness of the probe–vessel relationship during ultrasound localization. Second, real-time visualization of the needle tip relative to the vessel facilitated earlier correction of deviations and more accurate depth control during catheterization. Finally, the elimination of gaze shifts reduced the need for visual reorientation and cognitive integration of separate visual inputs, thereby streamlining the overall workflow. These findings are consistent with previous studies (12, 14) and further validate the utility of this system in a high-difficulty elderly population.

The overall incidence of adverse events in the smart glasses group (8.25%) was significantly lower than in the control group (21.65%; *p* = 0.009; RR = 0.381). Repeated punctures can damage the vascular endothelium, increasing the risk of hematoma, and can stimulate vascular smooth muscle, leading to vasospasm. In this study, the reductions in hematoma (8.25% vs. 14.43%) and catheter malfunction (1.03% vs. 7.22%) are consistent with the decreased number of puncture attempts. By improving first puncture success and reducing repeated punctures, integrated wireless ultrasound and smart glasses system may have contributed to the lower overall rate of short-term procedure-related adverse events. However, this study was not powered to detect differences in individual complications, and this finding should be interpreted cautiously.

Operator satisfaction was significantly higher in the smart glasses group than in the control group [86 (79–90) vs. 70 (60–79); *p* < 0.001]. The distribution of ergonomic fatigue was also more favorable in the smart glasses group, with a higher proportion of operators reporting no fatigue (69.07% vs. 27.84%). Anesthesiologists are prone to work-related musculoskeletal disorders due to continuous repetitive movements and unstable postures during procedures (27). Smart glasses effectively reduce the number of head rotations, allowing operators to maintain a neutral working posture and thereby decreasing the risk of musculoskeletal fatigue (14–16, 21).

The strengths of this study include the following: First, to our knowledge, it is the first randomized controlled trial specifically investigating the use of integrated wireless ultrasound and smart glasses system for radial artery catheterization in elderly patients (≥65 years). Second, hand–eye coordination was assessed using objective behavioral indicators. Third, this study is the first to integrate a wireless handheld color ultrasound probe, a portable AR console, and smart glasses into a unified puncture guidance system, offering greater portability compared with previous setups (12, 13). In contrast, the study by Gözen et al. relied on a tablet as the central image transmission device, which was less portable (13). Fourth, the wireless probe eliminates the limitations of conventional ultrasound cables, reducing the risk of procedural field contamination and better meeting infection control requirements. Fifth, all procedures were performed by three experienced anesthesiologists, with standardized placement of the ultrasound machine and operator positioning (based on operator ergonomic preference) and all operators being right-handed, thereby minimizing inter-operator variability.

The use of integrated wireless ultrasound and smart glasses system also has potential drawbacks. In this study, the Rokid Max 2 AR glasses weighed 75 g, and prolonged use caused pressure on the operator’s nose. Additionally, during the procedure, operators needed to switch their visual attention between the procedural field and the ultrasound image displayed in the smart glasses. Frequent eye refocusing can disrupt the alignment of sensory information, leading to dizziness and increased visual fatigue. Over time, this may result in pronounced ocular discomfort, including eye strain, dryness, foreign body sensation, and burning, as well as decreased near vision (28). Prolonged continuous use could affect both procedural comfort and stability.

Third, wireless video transmission may introduce delays, which could interfere with puncture and catheterization. Fourth, when multiple operators use smart glasses, there is a risk of cross-contamination, necessitating dedicated device disinfection procedures. Fifth, the battery life of smart glasses and the wireless ultrasound probe is considerably shorter than that of conventional ultrasound systems, requiring dedicated personnel to manage charging, maintenance, and disinfection.

### Limitations

This study has several limitations. First, it was a single-center study, which may limit the generalizability of the findings. Second, only the short-axis out-of-plane (SA-OOP) technique was used, and the long-axis in-plane approach was not evaluated; therefore, the study could not comprehensively compare the performance of different puncture techniques. Future studies incorporating multiple approaches may improve the applicability and comparability of the results. Third, postoperative ultrasound measurements were operator-dependent and may have introduced variability. Fourth, operator-reported outcomes may have been influenced by the lack of operator blinding and repeated measurements within the same operators (14). Although we attempted to minimize inter-operator variability through standardized training and balanced case allocation, the absence of statistical adjustment for clustering (e.g., mixed-effects modeling) may have led to underestimation of variance. Therefore, the findings should be interpreted with caution. Because the intervention (use of smart glasses) was visually apparent, blinding of behavioral outcome assessors was not feasible, which may have introduced measurement bias despite prospective recording and independent video verification. In addition, certain behavioral indicators, such as head rotations, are inherently linked to the intervention itself and should therefore be interpreted as process-related measures reflecting workflow changes rather than independent clinical outcomes. Fifth, all procedures were performed by experienced attending anesthesiologists; thus, the effectiveness of smart glasses in less-experienced operators remains unclear. Sixth, the use of smart glasses introduces a potential risk of latency between the ultrasound device and the display. Although no significant delay was observed in this study, this factor should be considered in future applications. Seventh, multiple secondary outcomes were analyzed without formal adjustment for multiplicity; therefore, these findings should be interpreted as exploratory and hypothesis-generating.

In addition, there is limited robust comparative literature on integrated wireless ultrasound combined with smart glasses for radial artery catheterization, which may restrict contextual interpretation of the present findings.

Another important limitation is that different ultrasound systems were used in the two groups. The intervention group employed a wireless handheld ultrasound device integrated with smart glasses, whereas the control group used a conventional console-based system. Therefore, this study evaluates the effect of the integrated system rather than isolating the independent contribution of smart glasses. Differences in device characteristics, including display modality, probe design, and ergonomics, may have influenced the results. Future studies using the same ultrasound platform with and without smart glasses are warranted to determine the independent effect of the smart glasses component.

## Conclusion

In elderly patients undergoing radial artery catheterization, integrated wireless ultrasound and smart glasses system improved first puncture success and reduced procedure-related behavioral indicators associated with hand–eye coordination compared with conventional ultrasound guidance. This approach also shortened procedure time and increased operator-reported satisfaction. Further studies are needed to clarify the independent contribution of each component and to validate these findings in broader clinical settings.

## Data Availability

The datasets presented in this study can be found in online repositories. The names of the repository/repositories and accession number(s) can be found at: https://osf.io/g6pbc/overview?view_only=098ba5f510cc4346b8d2af4f89b5a298.

## Footnotes

1 https://www.chictr.org.cn/showproj.html?proj=271966, ChiCTR2500103198; registration date: May 26, 2025
