# Supplementary material for: Smart glasses integrated with a wireless ultrasound system for radial artery catheterization in elderly patients: a randomized controlled trial
Source: Front Med (Lausanne). 2026 Jul 23;13:1884099. doi: 10.3389/fmed.2026.1884099 (PMC13442540; doi:10.3389/fmed.2026.1884099)
Supplement: Supplementary file 1 [file Table_1.docx]

**Supplementary Table S1. Categorical distribution of operator satisfaction scores**

| **Satisfaction score category** | **Smart glasses group (n = 97)** | **Control group (n = 97)** | **RR (95% CI)** |
| --- | --- | --- | --- |
| 0–40 | 0 (0.00%) | 0 (0.00%) | N/A |
| 41–60 | 7 (7.22%) | 25 (25.77%) | 0.280 (0.127, 0.617) |
| 61–80 | 29 (29.90%) | 54 (55.67%) | 0.537 (0.377, 0.764) |
| 81–100 | 61 (62.89%) | 18 (18.56%) | 3.389 (2.174, 5.283) |

Note: Data are presented as n (%). Category-specific relative risks with 95% confidence intervals are presented as descriptive effect estimates. The inferential analysis of satisfaction was performed using the continuous VAS score and is reported in Table 3. No operator in either group reported a satisfaction score of 0–40.
RR, relative risk; CI, confidence interval; VAS, visual analogue scale.
